# What is the effect of volunteer community responders network on out-of-hospital cardiac arrest outcomes in the swiss canton of fribourg? a five-year retrospective observational study

**DOI:** 10.1016/j.resplu.2025.101009

**Published:** 2025-06-19

**Authors:** Sébastien Pugnale, Ludovic Galofaro, Serban-George Puricel, Dorian Garin, Youcef Guechi, Stéphane Cook, Vincent Ribordy

**Affiliations:** a Department of Emergency Medicine, University and Teaching Hospital, Fribourg, Switzerland; b Department of Cardiology, University and Teaching Hospital, Fribourg, Switzerland; c Faculty of Science and Medicine, University of Fribourg, Switzerland

**Keywords:** Volunteer Community Responder, Out-of-Hospital Cardiac Arrest, Cardiopulmonary Resuscitation, Swiss Canton Fribourg, Clinical Outcomes, Survival to Hospital Discharge

## Abstract

**Aim:**

This study aims to assess the impact of the Volunteer Community Responders (VCR) Network on the outcomes of Out-of-hospital Cardiac Arrest (OHCA) patients in the Swiss Canton Fribourg from 2018 to 2022.

**Methods:**

We collected data from the SWISSRECA registry of all OHCA occurring in the Swiss Canton of Fribourg between January 1, 2018 and December 31, 2022. The patients were divided in two groups according to whether a VCR intervened and were compared using descriptive statistics. We constructed exact multivariable logistic regression models to analyse the association of a VCR intervention with Return of Spontaneous Circulation (ROSC) rate, survival to hospital admission and survival to hospital discharge, adjusting for age, sex, emergency services arrival time, witness status, initial rhythm and location of the OHCA.

**Results:**

We included 559 patients, 319 in the VCR intervention group and 240 in the other group. The key differences were more home located OHCA (80% vs 63%; p-value = < 0.001), longer EMS arrival times (13 [11, 17] vs 11 [8, 14] minutes; p-value = < 0.001) and more pre-EMS CPR performed (95% vs 63%; *p* < 0.001) and pre-EMS AED usage (48% vs 13%; *p* < 0.001) in the VCR intervention group. In the adjusted model, the odds ratios with VCR Cardiopulmonary Resuscitation (CPR) were 0.82 (95% CI: 0.39–1.74; *p* = 0.61) for ROSC, 0.65 (95% CI: 0.27–1.53; *p* = 0.32) for survival to hospital admission and 1.22 (95% CI: 0.26–5.74; *p* = 0.80) for survival to hospital discharge. In the sub-cohort of home-located OHCA, consisting of 192 patients the VCR intervention group and 202 in the other group, the adjusted odds ratios of VCR CPR were 0.78 (95% CI: 0.34–1.81; *p* = 0.57) for ROSC, 0.75 (95% CI: 0.27–2.08; *p* = 0.58) for survival to hospital admission and 1.34 (95% CI: 0.23–7.79; *p* = 0.75) for survival to hospital discharge.

**Conclusion:**

The rates of CPR performed and AED usage before EMS arrival were higher in the VCR intervention group. However, the VCR intervention was not independently associated with ROSC rate, survival to hospital admission and survival to hospital discharge. Potential key explanations are the sequential manual activation of the VCR network, leading firstly to potential delays in VCR deployment and secondly to potentially more VCR deployment on home-located OHCAs or on OHCA with longer expected EMS travel time. Updating the deployment system of the emergency medical communication centre could be a key improvement measure to address this. Further studies are needed to explore these issues and to improve the chain of survival including the VCR Network.

## Highlights

• First study of the Volunteer Community Responders Network in the Swiss Canton of Fribourg.
• Volunteer Community Responders intervention induced higher CPR rates and AED usage.
• Survival was not independently associated with Volunteer Community Responders intervention.
• Delayed arrival of Volunteer Community Responders due to sequential alarm activation was identified as a key factor.

## Introduction

Out-of-hospital cardiac arrest (OHCA) is a significant global health concern, with timely and high-quality cardiopulmonary resuscitation (CPR) being the cornerstone of improving patient outcomes.^1^ In pursuit of this objective, it is now widely recognized that implementing high quality national or international OHCA registries that collect standardized Utstein-style data can help to enhance performance and survival of OHCA patients.^2^, ^3^, ^4^ In Switzerland, the Swiss Canton Ticino led the way by establishing a regional web-based registry of OHCA, TI-RECA, in 2005.^5^ This registry has yielded precious information to improve the chain of survival,^5^, ^6^, ^7^, ^8^ allowing in particular the development of the Utstein-Based Return Of Spontaneous Circulation (UB-ROSC) score, which allow to determine the probability of sustained ROSC leading to hospital admission based on clinical variables of OHCA patients setting realistic expectations during CPR.^9^, ^10^ Building on this success, the Interassociation of Rescue expanded the effort to a national level by creating the Swiss registry of out-of-hospital cardiac arrest (SWISSRECA) in 2017, 11, 12 using the Utstein OHCA template.^2^, ^3^, ^13^

On a European level, the EuReCa studies, using European OHCA registries, have shown a wide variability in OHCA survival rates between countries, although unable to completely identify the factors responsible.^14^, ^15^ Similarly, the ESCAPE-NET group demonstrated an improved survival of OHCA patients in countries where a VCR network is implemented compared to countries without a VCR network, but again with a wide variability in the OHCA survival rates observed.^16^

To Improve OHCA patient outcomes in the Swiss Canton Fribourg the political authorities implemented a network of VCR trained in resuscitation and defibrillation in 2016 to complete the chain of survival based on system saving lives and previous experience,^5^, ^6^, ^7^, ^8^, ^17^, ^18^ and joined the SWISSRECA from 2018. To date, OHCA patients’ survival in the Swiss Canton of Fribourg and the impact of the VCR network on improving OHCA patients’ outcomes has not been assessed. The aim of this study was to assess the impact of the VCR intervention on survival rate of patients suffering OHCA compared to patients without VCR intervention.

## Methods

### Setting

The Swiss Canton of Fribourg (western Switzerland), count a growing population of 334′465 inhabitants (census year 2022) in an urban, rural and mountain area of 2701 km^2^ **(Fig. S1, supplementary materials)**.^19^ One emergency medical communication centre received all emergency calls. Dispatchers are mainly paramedics or healthcare professionals. Calls to the emergency medical communication centre are handled with standardized protocols to dispatch appropriate aid to medical emergencies. In all cases of suspected cardiac arrest (unconscious and not breathing), systematized caller interrogation (Medical Priority Dispatch System^TM^) and pre-arrival telephone-CPR instructions are provided as standard. At the time of the study, the incidence of OHCA treated by emergency medical services (EMS) was 71,6 per 100′000 persons. The management of OHCA is first handled by five local EMS units strategically positioned to cover the entire region and activation is based on a 2-tier system. This includes an ambulance with paramedics skilled in advanced cardiovascular life support and, when necessary, a ground or helicopter-based team including an emergency physician. In cases of suspected cardiac arrest, interventions systematically involve a paramedic and an emergency physician based team.^20^, ^21^, ^22^

### First responders network

**Fig. 1:**
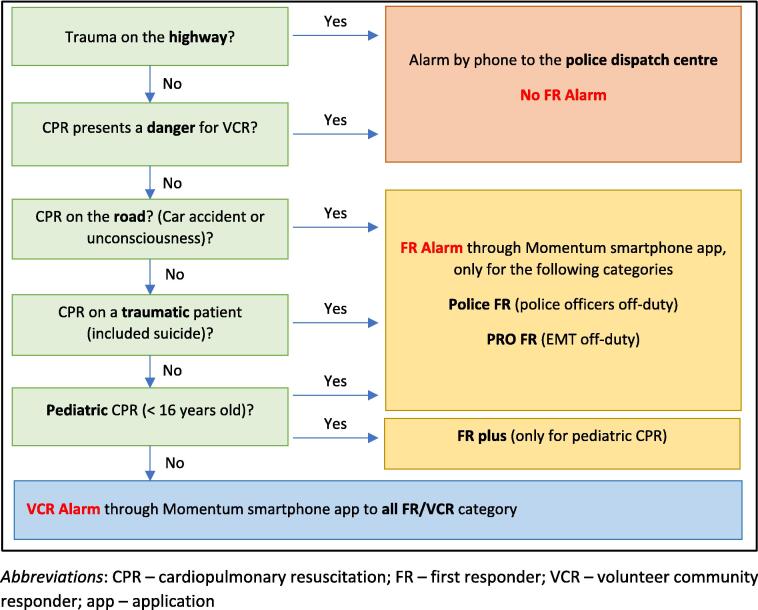
Criteria for a VCR to be dispatched to an OHCA by the EMCC in the SCF**.***Abbreviations*: CPR – cardiopulmonary resuscitation; FR – first responder; VCR – volunteer community responder; app – application The emergency call centre of the SCF applies these criteria in each case of OHCA to decide whether to activate the FR network and, if so, which categories are dispatched. Translated and adapted from the internal procedure for the SCF of the CASU 144 with permission of Manuela Spicher, head of the CASU 144, version 3, 21.11.2018.

To improve the probability of survival for OHCAs, a “chain of survival” concept has gradually been deployed in the Swiss Canton of Fribourg. Since 2016, a network of first responders comprises VCR and police patrols who were trained in CPR and Automated External Defibrillator (AED) use. Main organisational and system characteristics are very similar to the system described previously in Ticino, Switzerland.^5^, ^6^, ^7^, ^8^ Eligibility for VCR status requires being more than 18 years old, completing an official BLS-AED course recognized by the Swiss resuscitation council, and maintaining up-to-date training every 2 years. VCR sign the VCR Charter outlining their commitment framework. The VCR Foundation on behalf of the Swiss Canton Fribourg state is responsible for the management of training and staffing levels of VCR and for the AED fleet location. In case of a suspected OHCA, if dispatch criteria (Fig. 1) are met with exclusions of trauma, unsafe situations, and children < 8-year-old, the nearest VCRs are alerted via a smartphone app (Momentum, First Responder JU & FR) 24 h a day. Activation of the network is sequential and manual: the emergency medical communication centre coordinator first manually fills in the EMS deployment system, then, once the EMS has been deployed, manually fills in the Momentum system and sends the app alert to the VCRs. Once they confirm their availability, VCRs receive the patient’s and nearest AED’s location.^18^ Their mission is to provide initial care following the BLS AED guidelines of the Swiss resuscitation council during the critical first 10 min while waiting for the EMS, thus playing a vital role in the chain of survival. The number of VCRs and AEDs evolved during the study period reaching about 1621 to 2050 VCRs and 440 to 600 AEDs.

### Study design and population

**Fig. 2:**
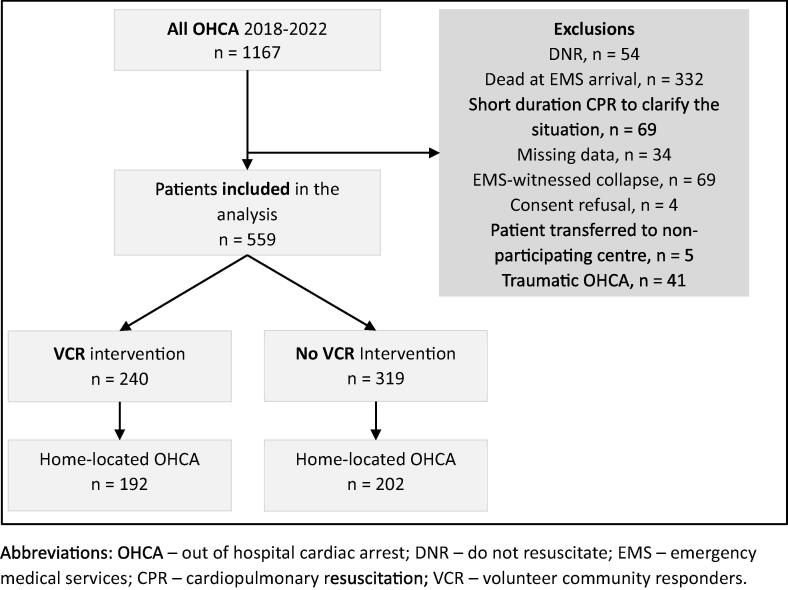
Cases Flowchart. Abbreviations: OHCA – out of hospital cardiac arrest; DNR – do not resuscitate; EMS – emergency medical services; CPR – cardiopulmonary resuscitation; VCR – volunteer community responders.

We performed a retrospective observational analysis using data from the SWISSRECA. The study included a consecutive sample of adults (≥18 years old) experiencing OHCA between January 1, 2018 and December 31, 2022 in the Swiss Canton of Fribourg. The following patients were excluded from the analysis: patients with incomplete data, patients with “do not resuscitate” order, OHCAs of traumatic etiology, when collapse was witnessed by EMS with immediate ROSC, patients declared dead upon EMS arrival and those who objected to the use of their data (Fig. 2).

### Study endpoints

The primary endpoint was survival to hospital discharge. The secondary endpoints were ROSC rate and survival to hospital admission.

## Data collection and definitions

Anonymised data were sourced and coded from the SWISSRECA and stored in a secure web application for building and managing online surveys and databases, Research Electronic Data Capture (REDCap). The SWISSRECA has already been described in detail.^12^, ^23^, ^24^ The main characteristics and the evolutions of the VCRs’ network were extracted from the annual report of the First Responders JU & FR Foundation and are listed in Table 1. Data on the VCR network activation time and by extension the delay time to system activation as well as the delay time between activation of the network and the arrival of the VCR on scene are not currently collected by SWISSRECA, as the standards for uniform reporting of first responder systems have only recently been published and this data has only been added to the Utstein template since the last update dating from 2024.^2^, ^3^, ^13^, ^25^

**Table 1:** Setting and evolution of characteristics of the VCRs’ network during the study period.

**Characteristics**	**2018**	**2019**	**2020**	**2021**	**2022**	**Mean**
Population	318′717	321′783	325′496	329′809	334′465	326′054
Area (km^2^)	2701	−	−	−	−	2701
AEDs/km^2^	0,16	−	−	−	−	0,22
AED/100′000	138	−	−	−	179,4	158,7
OHCA	225	226	228	243	246	234
OHCA incidence	70,6	70,2	70	73,7	73,6	71,6
Number of VCR	1850	2400	2300	2340	2050	2188
VCR/km2	0,68	0,86	0,85	0,86	0,76	0,8
VCR/population (%)	0,58	0,74	0,7	0,71	0,61	0,67
Overall VCR activation (attendance rate of VCR activation %)	136 *	176 *	128 *	243 *	239 (81,7)	184,4 (87)
Interval from application alert to VCR arrival on scene (min.; mean)	5,5	5,9	5,15	5,9	5,6	5,7

Abbreviations: OHCA – out-of-hospital cardiac arrest; SCF – swiss canton fribourg; VCR – volunteer community responders. Attendance rate of VCR activation is the ratio between the actual VCR interventions number and the total number of VCR activations (* not available in % from 2018 to 2021). Interval from application alert to VCR arrival on scene (min.; mean) is the delay between the activation of the VCR network by the EMCC through an alert sent in the Momentum app and the time of VCR arrival on scene determined by the Momentum App system.^8^, ^9^, ^10^

Data were defined and reported as follows: *VCR intervention* was classified as “yes” if the VCR network was activated with at least one VCR answering the alert and arriving on scene, and “no” otherwise. *VCR CPR performed* was classified as “yes” if the VCR initiated or continued the BLS procedure before EMS arrival, and “no” otherwise. *VCR AED usage* was classified as “yes” if the VCR initiated or helped the use of the AED before EMS arrival and “no” otherwise. *Pre-EMS CPR* was defined as the initiation of the CPR before the EMS arrival either by the bystander or the VCR. *Pre-EMS AED usage* was defined as the use of an AED before the EMS arrival, either by the bystander or by the VCR, regardless of whether a shock was delivered. *Initial rhythm* was classified as either “shockable” (ventricular fibrillation and pulseless ventricular Tachycardia) or “non-shockable” (asystole, pulseless electrical activity). *Collapse to BLS time* was defined as the interval from the time of collapse to the initiation of the BLS procedures. *Call to BLS time* was defined as the interval from the time of the emergency call to the initiation of the BLS procedure. For the rest of the variables, the definitions established in the 2024 Utstein style uniform reporting guidelines were used.^2^, ^3^, ^13^

### Timing data

The timing data are collected retrospectively in the following way before being integrated into the SWISSRECA registry: *Call at the emergency medical communication centre time* is recorded automatically in the emergency medical communication centre software at the time the call is taken, the *Bystander CPR initiation time* is estimated by the bystander if CPR began before the call, the *VCR network activation time* is recorded by the Momentum application as soon as the emergency medical communication centre coordinator sends the alert. The *VCR arrival time on scene* is automatically recorded by the Momentum app as soon as the VCR's geographical position matches that of the OHCA. The *VCR CPR initiation time* is estimated by the VCR and then collected by the EMS. The *First Defibrillation time* is estimated by the bystander or by the VCR and then collected by the EMS. The *EMS arrival time on scene* is collected automatically by the EMS computer system, as soon as its geographical position matches that of the OHCA.

### Statistical analysis

Patients were classified into two groups based on VCR intervention. Baseline characteristics were compared using univariate statistical tests. Continuous variables were described using the mean and standard deviation (Gaussian distribution) or the median and interquartile range (other distributions), depending on the distribution, which was analysed using quantile–quantile diagrams and the Shapiro-Wilk test. Categorical variables were presented as absolute and relative frequencies. For continuous variables with normal distribution, the *t*-test was used; for non-normal distributions, the Wilcoxon (Mann-Whitney) test was applied. Categorical variables were compared using the χ2 or Fisher test, based on expected frequencies.

Two multivariate exact logistic regression models using penalized maximum likelihood estimation (Firth’s method) to address potential separation or small-sample bias, were constructed to investigate the association between the mode of CPR and each binary outcome of interest (survival to hospital discharge, survival to hospital admission, and ROSC rate). The primary independent variable was a four-level categorical indicator of CPR type (“No CPR,” “Bystander only,” “VCR only,” or “Bystander + VCR”). The first model contained no additional variables. The second model was adjusted for age, sex, location of OHCA, witness status of OHCA, initial rhythm and time to EMS arrival, reflecting the data likely available at initial management. We reported odds ratios (OR) and corresponding 95% confidence intervals (CI), as well as p-values for significance testing. Sensitivity analyses were performed to assess the robustness of our findings. A sensitivity analysis was conducted by including and excluding independent variables in different models: a “model alone”, a “model with age and gender”, and another “model with independent variables” such as Home-located cardiac arrest and Time to EMS arrival. Missing values were dropped listwise. The same analyses were conducted for the subgroup of home-located OHCA.

Analyses were conducted using Python (version 3.13.0) and R (version 4.4.2), utilizing the logistf package (version 1.26.0) via Python’s rpy2 interface (version 3.5.17). In Python, we used NumPy (version 1.24.3), Pandas (version 2.0.1), psycopg2 (version 2.9.10), statsmodels (version 0.14.4), and SciPy (version 1.15.2). Analyses were conducted in Visual Studio Code (version 1.95, © Microsoft 2025) with a local PostgreSQL (version 14.15) using DBeaver version 24.3.4 (© DBeaver Corp 2025) as the data management source.

### Ethical review

The study complied with the Declaration of Helsinki and was approved by the local ethics committee (Commission cantonale d’éthique de la recherche sur l’être humain − CER-VD) at the University and Teaching Hospital Fribourg (protocol no. 2023–02366). For all patients deceased at the time of the study, the ethics committee granted approval to reuse their data with a waiver of consent. For all living patients, written consent was obtained.

## Results

### Patients

During the study period, 1,167 OHCAs occurred in the Swiss Canton Fribourg. The overall incidence remained relatively stable over the years (71.6 ± 1.9 OHCA per 100′000 inhabitants, p-value = 0.96) (Table 1). After applying the exclusion criteria 559 cases were included in the final analyses. Patients and resuscitation characteristics are presented for the whole cohort in Table 2 and for the sub-cohort of home located OHCA in **Table S1** (supplementary materials).

**Table 2:** Characteristics of and statistical comparisons between the group without VCR intervention and with VCR intervention in the whole cohort.

**Characteristics (cohort)**	**All cases**	**Without VCR intervention**	With VCR intervention*	**p-value**
OHCA, n (%)	559 (1 0 0)	319 (57)	240 (43)	NA
Age, years, median [IQR]	70 [59, 79]	70 [58, 79]	70 [61, 79]	0.5
Male, n (%)	421 (75)	234 (73)	187 (78)	0.2
Home located, n (%)	394 (70)	202 (63)	192 (80)	< 0.001
Bystander witnessed collapse, n (%)	317 (57)	197 (62)	120 (50)	0.005
Bystander CPR performed, n (%)	362 (65)	201 (63)	161 (67)	0.3
Bystander AED usage, n (%)	51 (9.1)	40 (13)	11 (4.6)	< 0.001
Call assisted CPR performed, n (%)	367 (66)	172 (54)	195 (81)	< 0.001
VCR CPR performed, n (%)	−	−	194 (81)	NA
VCR AED usage, n (%)	−	−	112 (47)	NA
Pre-EMS CPR performed, n (%)	429 (77)	201 (63)	228 (95)	< 0.001
Pre-EMS AED usage, n (%)	155 (28)	40 (13)	115 (48)	< 0.001
Initial rhythm shockable, n (%)	164 (29)	81 (25)	83 (35)	0.018
Collapse to BLS time, minutes, median [IQR]	5 [1, 10]	5 [1, 10]	5 [2, 9]	0.8
Call to BLS time, minutes, median [IQR]	2 [0, 8]	3 [0, 9]	2 [0, 6]	0.016
Call to first Defibrillation time, minutes, median [IQR]	13 [9, 17]	13 [9, 17]	13 [10, 17]	0.8
Call to VCR arrival time, minutes, median [IQR]	−	−	8 [5, 12]	NA
Time to EMS arrival on scene, minutes, median [IQR]	12 [9, 16]	11 [8, 14]	13 [11, 17]	< 0.001
UB-ROSC score derived probability of sustained ROSC, percentage, median [IQR]	12 [6, 29]	13 [9, 37]	10 [6, 18]	< 0.001
ROSC achieved, n (%)	178 (32)	116 (36)	62 (26)	0.008
Survival, n (%)				
- To hospital admission	153 (27)	102 (32)	51 (21)	0.005
- To hospital discharge	43 (7.7)	32 (10)	11 (4.6)	0.017
CPC score at discharge, n (%)				> 0.9
- 1	26 (4.6)	18 (5.6)	8 (3.3)	
- 2	11 (2)	9 (2.8)	2 (0.8)	
- 3	5 (0.9)	4 (1.3)	1 (0.4)	
- Unknown	1 (0.2)	1 (0.3)		

Abbreviations: OHCA – out-of-hospital cardiac arrest; CPR – cardiopulmonary resuscitation; AED – automated external defibrillator; VCR – volunteer community responders; EMS – emergency medical services; BLS – basic life support; IQR – interquartile range; ROSC – return of spontaneous circulation; ED – emergency department; CPC – cerebral performance category; NA – not applicable.

* *VCR intervention* indicates that the VCR network was activated with at least one VCR answering the alert and arriving on scene of the OHCA.

### VCR interventions

During the study period, the VCR network was activated in 293 of the 559 cases included in the analyses, with a VCR responding to the alert, being dispatched and arriving at the scene in 240 of these cases (response and attendance rate = 81.9%). Most of those missions (137; 57%), took place from 08 *h*00 to 18 *h*00, 44 (18%) between 18 *h*00 to 22 *h*00 and 59 (25%) during the night. The mean first responder arrival time was 5.66 min (40% < 5 min, 58,4% between 5–15 min and 1.6% > 15 min). Cases without VCR intervention (319; 57%) and those with VCR intervention (240; 43%) were compared. Patient and resuscitation characteristics are presented in Table2. Differences were observed between the two groups. In the group with VCR intervention there were more home located-OHCA (80% vs 63%; p-value = < 0.001), less collapse witnessed (50% vs 62%; *p* = 0.005), more call assisted CPR performed (81% vs 54%; p value = < 0,001), more pre-EMS CPR performed (95% vs 63%; p value = < 0.001), more pre-EMS AED usage (48% vs 13%; p value = < 0.001) and more initial rhythm shockable (35% vs 25%; p value = 0.018). Time until EMS arrival was longer in the group with VCR intervention (13 [11, 17] vs 11 [8, 14] minutes; p-value = < 0.001), and the median UB-ROSC score derived probability of sustained ROSC was better in the group without VCR intervention (13% [9, 37] vs 10% [6, 18]; p-value = < 0.001).

### Observational outcomes

Outcome comparisons between the group without VCR intervention and with VCR intervention respectively showed differences in ROSC achievement rate (36% vs 26%; *p* = 0.008), survival to hospital admission (32% vs 21%; *p* = 0.005) and survival to hospital discharge (10% vs 4.6%; *p* = 0.017) and demonstrated an inverse association with VCR intervention. The results of the exact logistics regressions models for the whole cohort are presented in Table 3 for the primary outcome and in supplementary materials (**Table S2** and **Table S3**) for the secondary outcomes.

**Table 3:** Results of the two exact logistic regressions models for the primary outcome (survival to hospital discharge) for all cases. Model 1 is unadjusted, Model 2 is adjusted with age, gender, home location of the OHCA, time to EMS arrival, initial rhythm and witness status of the OHCA.

	**Co-variables**	**OR**	**95% CI**	**p-value**
**Model 1**	No pre-EMS CPR (reference)	1	−	−
	Bystander CPR only	2.77	1.11–6.95	0.03
	VCR CPR only	0.74	0.18–3.04	0.67
	Bystander and VCR CPR	0.98	0.33–2.89	0.97
**Model 2**	No pre-EMS CPR (reference)	1	−	−
	Bystander CPR only	1.83	0.65–5.16	0.26
	VCR CPR only	1.22	0.26–5.74	0.80
	Bystander and VCR CPR	0.89	0.26–3.06	0.86
	Age	0.99	0.97–1.01	0.40
	Male gender	1.02	0.42–2.49	0.96
	Home located cardiac arrest	0.43	0.21–0.92	0.029
	Time to EMS arrival	0.89	0.82–0.97	0.006
	Initial rhythm shockable	5.28	2.49–11.19	< 0.001
	Witnessed OHCA	5.80	2.14–15.76	< 0.001

Abbreviations: CPR – cardiopulmonary resuscitation; VCR – volunteer community responders; EMS – emergency medical services; OHCA – out-of-hospital cardiac arrest; OR – odds ratio; CI – confidence interval.

When restricted to the home-located OHCA, outcomes comparisons between the group without VCR intervention and with VCR intervention respectively showed no differences in ROSC achievement rate (30% vs 24%; *p* = 0.2), survival to hospital admission (22% vs 19%; *p* = 0.5), and survival to hospital discharge (5.4% vs 3.6%; *p* = 0.4). The results of the exact logistics regressions models for the subgroup of home located OHCA are presented in Table 4 for the primary outcome and in supplementary materials (**Table S4** and **Table S5**) for the secondary outcomes. Sensitivity analyses were not significantly different in the adjusted and unadjusted models (Table 3 and Table 4).

**Table 4:** Results of the two exact multivariate logistic regressions models for the primary outcome (survival to hospital discharge) for the subgroup of home-located OHCA. Model 1 is unadjusted, Model 2 is adjusted with age, gender, time to EMS arrival, initial rhythm and witness status of the OHCA.

	**Co-variables**	**OR**	**95% CI**	**p-value**
**Model 1**	No pre-EMS CPR (reference)	1	−	−
	Bystander CPR only	2.33	0.60–9.04	0.22
	VCR CPR only	1.37	0.27–7.00	0.71
	Bystander and VCR CPR	0.99	0.22–4.53	0.99

**Model 2**	No pre-EMS CPR (reference)	1	−	−
	Bystander CPR only	1.70	0.40–7.16	0.47
	VCR CPR only	1.34	0.23–7.79	0.75
	Bystander and VCR CPR	0.62	0.12–3.22	0.57
	Age	0.98	0.95–1.01	0.24
	Male gender	0.60	0.21–1.74	0.35
	Time to EMS arrival	0.98	0.88–1.09	0.72
	Initial rhythm shockable	5.86	2.00–17.21	0.001
	Witnessed OHCA	3.65	0.96–13.88	0.06

Abbreviations: CPR – cardiopulmonary resuscitation; VCR – volunteer community responders; EMS – emergency medical services; OHCA – out-of-hospital cardiac arrest; OR – odds ratio; CI – confidence interval.

## Discussion

This observational study based on the SWISSRECA evaluated the impact of VCR intervention on survival and ROSC rate following OHCA. The overall survival to hospital discharge was 7.5%, lower than the European average (10%),^14^, ^15^ although survival varies widely between geographic regions and health systems (0.6% to 25%).^26^, ^27^ While advanced interventions have limited effect on survival with good neurological outcomes,^28^ early high quality bystander-CPR and defibrillation,^29^ as the implementation of VCR networks have been associated with improved outcomes.^30^, ^31^, ^32^ Our results highlighted a key increased rate of pre-EMS CPR and pre-EMS AED usage in the VCR intervention group. However, we did not identify a statistically significant association between VCR CPR and survival to hospital discharge neither in the whole cohort nor in the subgroup of home-located OHCAs.

Several factors may have confounded the results. First, more OHCA took place at home in the VCR intervention group (80% vs 63%; *p* < 0.001) which probably led to the less witnessed OHCA (50 vs 62%; *p* = 0.005),^33^ delaying the initiation of the CPR, and more telephone instructed CPR performed by elderly bystander, which could have impaired the quality of the CPR.^34^ Second, the bystander AED usage was lower in the VCR intervention group (4.6% vs 13%; *p* = 0.001), which probably led to a delay to the first defibrillation. We hypothesised that this is a corollary to the higher proportion of home-located OHCA in this group, as the density of AED is lower in residential area,^33^ or due to OHCA happening in an area with a lower density of AED. Third, the delay to EMS arrival was longer in the VCR intervention group suggesting that more OHCA occurred in remote areas in this group.^35^, ^36^ According to these differences, the UB-ROSC score derived probability of sustained ROSC, median percentage, [IQR], was lower in the VCR intervention group (13% [9, 37] vs 10% [6, 18]; p-value = < 0.001), highlighting the worse prognosis of the cases in this group. On the other hand, there were more initial shockable rhythms in the group with VCR intervention, a hypothesised corollary of the higher pre-EMS AED usage in this group, which potentially biased the impact of the VCR intervention on outcomes.^37^

Comparison with the Swiss Canton of Ticino, national leader in VCR deployment and OHCA registry implementation,^5^, ^7^, ^9^, ^10^, ^38^ as well as geographically similar to the Swiss Canton Fribourg, revealed multiple key differences: first, EMS in the Swiss Canton Fribourg took longer to arrive on scene (12 [9, 16] vs 10 [8, 14] minutes),^5^, ^6^, ^7^, ^8^ hypothetically due to a mismatch between the geographical distribution of EMS and that of areas with a high incidence of OHCA within the Swiss Canton Fribourg. Second, OHCA are less witnessed (50% vs 69%), potentially due to more home-located OHCAs (65% vs 80%), which inherently worsens the prognosis.^7^ Last, as VCR arrival prior to EMS arrival determined a higher survival rate,^39^ our longer VCR time of arrival (8 [5, 12] vs 3.5 [2.8, 5.2] minutes) may be one of the key factors explaining a limited rate of VCR arrival prior to EMS, which led to no VCR CPR performed in 48 cases, no difference in defibrillation delay (13 [9, 17] vs 13 [10, 17] minutes; *p* = 0.8) and thus the lack of benefit from the VCR interventions.^38^, ^40^

The sequential activation process – first dispatching the EMS, then activating the VCRs’ network, due to an older version of the deployment software (SAGA System) – may have introduced several biases. First, given that the median delay from call to VCR arrival on scene is 8 min (Table 2) and that the mean delay from VCR network activation and VCR arrival on scene is 5.7 min, we hypothesise a systematic delay of the VCRs’ network activation of around 2 to 3 min. Second, although the VCRs’ network activation criteria doesn’t comprise the expected EMS travel time or the location of the OHCA, the aforementioned activation delay of 2 to 3 min may lead the coordinator to activate the VCR network preferentially in cases where the expected EMS travel time is longer or on home-located OHCAs, consequently involving the VCRs in cases with a poorer prognosis.^33^, ^35^, ^36^ Our hypotheses were supported by our data (**Table S6**, supplementary material) showing that the VCR network was more activated on home-located OHCA (58% vs 82%; *p* < 0.001) and when the initial rhythm was shockable (24% vs 34%; *p* = 0.02), and less activated when the bystander used the AED (14% vs 5%; *p* < 0.001), as well as when the OHCA was not witnessed (61% vs 53%; *p* = 0.046). Noteworthy, the VCR network was more activated when the EMS took more than 10 min to arrive on scene. Due to the precarious financial situation of the state of Fribourg since the Momentum app deployment, the political authorities were unable to allocate the necessary funds to update the deployment system, although this could be a key improvement to address these biases to allow VCR to be deployed automatically and at the same time as EMS in OHCA thus improving the impact of the VCR intervention.

Additional factors such as AED accessibility, VCR network coverage (number, density, repartition and availability), skills (VCR with basic or advanced life support competences), involvement of professional first responders (i.e. on–duty policemen) may also be relevant.^41^ In our study the spatial AED accessibility as well the VCR density and availability were not studied. Some authors indicate a 1% VCR coverage threshold and a favourable AED to VCR ratio from 1:2 to 1:3.^41^, ^42^ Future studies focusing on EMS, AED and VCR distribution compared to OHCA incidence should enable to optimise the distribution of resources in the Swiss Canton Fribourg territory and thus improve the survival of OHCA patients.

## Limitations

Our study should be interpreted in the context of the following limitations. It was a single-centre observational study, limiting generalisability. Second, the methodology – an analysis between two non-paired cohorts looking for effect of VCR intervention, may have introduced a bias by associating. We were unable to include patients treated directly by Helicopter EMS (54 cases, 4.4%), those directly transported to a non-participating centre and survivors excluded for refusal of reuse of their data. We are aware that this has lowered survival rates and perhaps biased the calculated impact of the VCR intervention. Our exclusion criteria may have statistically decreased survival rates on one hand (OHCA witnessed by the EMS) and statistically increased survival rates on the other hand (cases with a do-not-resuscitate order, cases declared dead upon EMS arrival). Standard reporting recommendations of VCRs’ network had only recently been published, thus limiting comparability with prior study due to heterogeneous data.^25^ Although the Utstein recommendations for uniform reporting precisely define each variable,^2^, ^3^, ^13^ timing variables such as Collapse to BLS time, Call to BLS time, Call to First Defibrillation time intervals, remain subject to recall and system-entry bias, which may have biases our results. On the other hand, this is the first study in the Swiss Canton Fribourg including more than 1000 consecutive OHCA during an ongoing VCR network deployment.

## Conclusions

The intervention of a VCR in case of OHCA was not independently associated with ROSC rate, survival to hospital admission or survival to hospital discharge, either in the whole cohort or in the sub-cohort of home-located OHCA. However, there were more pre-EMS CPR performed and more pre-EMS AED usage in the VCR intervention group. Potential key explanations are the sequential manual activation of the VCRs’ network, leading firstly to potential delays in VCR deployment and secondly to potential VCR deployment on home-located OHCAs or on OHCA with longer expected EMS travel time. Updating the deployment support system to allow VCR to be deployed automatically and at the same time as EMS in OHCA cases should make it possible to address these potential confounding factors. Further studies are however needed to explore the reasons for these findings and improve the VCR activation system and the network in the Swiss Canton Fribourg.

## CRediT authorship contribution statement

**Sébastien Pugnale:** Writing – review & editing, Writing – original draft, Visualization, Validation, Software, Resources, Project administration, Methodology, Investigation, Formal analysis, Data curation, Conceptualization. **Ludovic Galofaro:** Writing – review & editing, Visualization, Validation, Resources, Project administration, Investigation, Conceptualization. **Serban-George Puricel:** Writing – review & editing, Methodology, Formal analysis. **Dorian Garin:** Writing – review & editing, Software, Methodology, Formal analysis. **Youcef Guechi:** Writing – review & editing, Supervision. **Stéphane Cook:** Writing – review & editing, Validation, Supervision, Resources, Project administration, Methodology, Conceptualization. **Vincent Ribordy:** Writing – review & editing, Visualization, Validation, Supervision, Resources, Project administration, Methodology, Funding acquisition, Conceptualization.

## Declaration of competing interest

The authors declare that they have no known competing financial interests or personal relationships that could have appeared to influence the work reported in this paper.
