# Supplementary material for: What is the effect of volunteer community responders network on out-of-hospital cardiac arrest outcomes in the swiss canton of fribourg? a five-year retrospective observational study
Source: Resusc Plus. 2025 Jun 19;25:101009. doi: 10.1016/j.resplu.2025.101009 (PMC12270694; doi:10.1016/j.resplu.2025.101009)
Supplement: Supplementary Data 1 [file mmc1.docx]

**Figure S1**. Geographical location of the canton of Fribourg in Switzerland (population of 334’465 persons in 2022 and 2672 km^2^). Adapted from wikipedia.com.


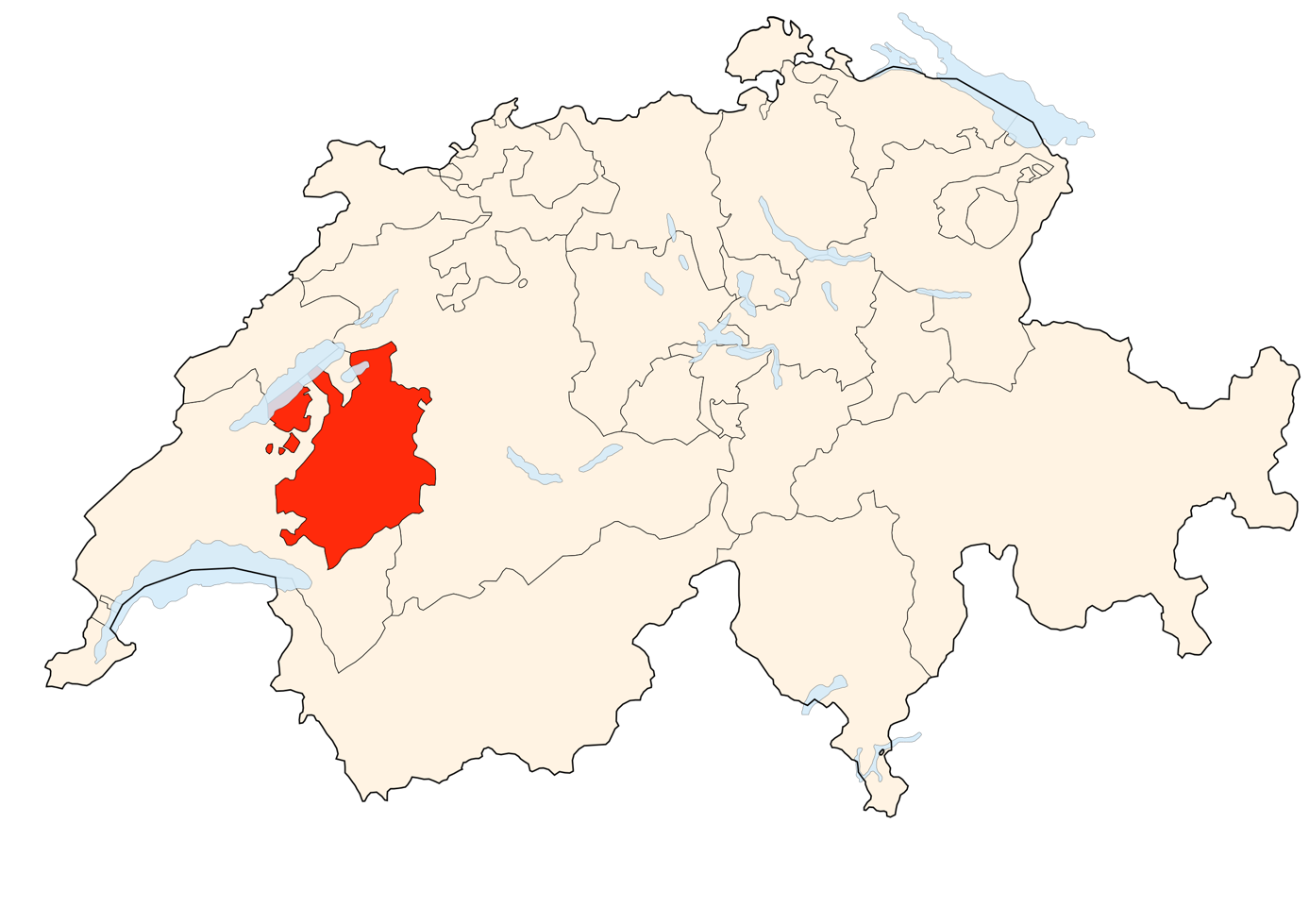


**Table S1.** Characteristics of and statistical comparisons between the group without VCR intervention and with VCR intervention in the sub-cohort of home-located OHCA.

| **Characteristics (cohort of home located OHCA)** | **All cases** | **Without VCR intervention** | **With VCR intervention*** | **p-value** |
| --- | --- | --- | --- | --- |
| OHCA, n (%) | 394 (100) | 202 (51) | 192 (49) | NA |
| Age, years, median [IQR] | 71 [61, 79] | 72 [60, 79] | 70 [61, 79] | > 0.9 |
| Male, n (%) | 290 (74) | 143 (71) | 147 (77) | 0.2 |
| Bystander witnessed collapse, n (%) | 223 (57) | 124 (61) | 99 (52) | 0.049 |
| Bystander CPR performed, n (%) | 234 (59) | 110 (54) | 124 (65) | 0.041 |
| Bystander AED usage, n (%) | 11 (2.8) | 6 (3.0) | 5 (2.6) | 0.8 |
| Call assisted CPR performed, n (%) | 256 (65) | 99 (49) | 157 (82) | < 0.001 |
| VCR CPR performed, n (%) | - | - | 155 (81) | NA |
| VCR AED usage, n (%) | - | - | 84 (44) | NA |
| Pre-EMS CPR performed | 391 (74) | 110 (54) | 181 (94) | < 0.001 |
| Pre-EMS AED usage, n (%) | 90 (23) | 6 (3.0) | 84 (44) | < 0.001 |
| Initial rhythm shockable, n (%) | 103 (26) | 40 (20) | 63 (33) | 0.003 |
| Collapse to BLS time, minutes, median [IQR] | 5 [2, 11] | 7 [2, 11] | 5 [2, 10] | 0.3 |
| Call to BLS time, minutes, median [IQR] | 3 [1, 9] | 6 [1, 10] | 2 [0, 7] | < 0.001 |
| Call to first Defibrillation time, minutes, median [IQR] | 15 [12, 18] | 16 [13, 21] | 14 [11, 18] | 0.026 |
| Call to VCR arrival time, minutes, median [IQR] | - | - | 9 [6, 12] | NA |
| Time to EMS arrival on scene, minutes, median [IQR] | 12 [9, 16] | 11 [8, 15] | 13 [11, 18] | < 0.001 |
| UB-ROSC score derived probability of sustained ROSC, percentage, median [IQR] | 10 [6, 18] | 12 [8, 20] | 9 [6, 13] | 0.001 |
| ROSC achieved, n (%) | 106 (27) | 60 (30) | 46 (24) | 0.2 |
| Survival, n (%) | | | | |
| - To hospital admission | 82 (21) | 45 (22) | 37 (19) | 0.5 |
| - To hospital discharge | 18 (4.6) | 11 (5.4) | 7 (3.6) | 0.4 |
| CPC score at discharge, n (%) |  |  |  | 0.8 |
| - 1 | 11 (2.7) | 6 (3.0) | 5 (2.6) |  |
| - 2 | 5 (1.3) | 3 (1.5) | 2 (1.0) |  |
| - 3 | 2 (0.5) | 2 (1.0) | - |  |

*Abbreviation*s: OHCA – out-of-hospital cardiac arrest; CPR – cardiopulmonary resuscitation; AED – automated external defibrillator; VCR – volunteer community responders; EMS – emergency medical services; BLS – basic life support; IQR – interquartile range; ROSC – return of spontaneous circulation; ED – emergency department; CPC – cerebral performance category.

**VCR intervention* indicates that the VCR network was activated with at least one VCR answering the alert and arriving on scene of the OHCA.

**Table S2**. Multivariate exact logistic regressions model for ROSC achieved (secondary outcome) for all cases. Model 1 is unadjusted, Model 2 is adjusted with age, gender, home location of the OHCA, time to EMS arrival, initial rhythm and witness status of the OHCA.

|  | **Co-variables** | **OR** | **95% CI** | **p-value** |
| --- | --- | --- | --- | --- |
| **Model 1** | No pre-EMS CPR (reference) | 1 | - | - |
|  | Bystander CPR only | 1.81 | 1.11 – 2.96 | 0.018 |
|  | VCR CPR only | 0.60 | 0.30 – 1.21 | 0.15 |
|  | Bystander and VCR CPR | 1.06 | 0.63 – 1.80 | 0.82 |
|  |  |  |  |  |
| **Model 2** | No pre-EMS CPR (reference) | 1 | - | - |
|  | Bystander CPR only | 1.49 | 0.87 – 2.55 | 0.14 |
|  | VCR CPR only | 0.82 | 0.39 – 1.74 | 0.61 |
|  | Bystander and VCR CPR | 1.06 | 0.59 – 1.91 | 0.84 |
|  | Age | 0.99 | 0.98 – 1.00 | 0.10 |
|  | Male gender | 0.56 | 0.36 – 0.87 | 0.009 |
|  | Home located cardiac arrest | 0.57 | 0.37 – 0.88 | 0.011 |
|  | Time to EMS arrival | 0,96 | 0.92 – 0.99 | 0.024 |
|  | Initial rhythm shockable | 2.34 | 1.54 – 3.57 | < 0.001 |
|  | Witnessed OHCA | 3.07 | 2.00 – 4.70 | < 0.001 |

*Abbreviation*s: CPR – cardiopulmonary resuscitation; VCR – volunteer community responders; EMS – emergency medical services; OHCA – out-of-hospital cardiac arrest; OR – odds ratio; CI – confidence interval.

**Table S3**. Multivariate binary logistic regressions model for survival to hospital admission (secondary outcome) for all cases. Model 1 is unadjusted, Model 2 is adjusted with age, gender, home location of the OHCA, time to EMS arrival, initial rhythm and witness status of the OHCA.

|  | **Co-variables** | **OR** | **95% CI** | **p-value** |
| --- | --- | --- | --- | --- |
| **Model 1** | No pre-EMS CPR (reference) | 1 | - | - |
|  | Bystander CPR only | 1.87 | 1.12 – 3.12 | 0.016 |
|  | VCR CPR only | 0.47 | 0.21 – 1.02 | 0.057 |
|  | Bystander and VCR CPR | 1.10 | 0.63 – 1.91 | 0.74 |
|  |  |  |  |  |
| **Model 2** | No pre-EMS CPR (reference) | 1 | - | - |
|  | Bystander CPR only | 1.33 | 0.75 – 2.36 | 0.34 |
|  | VCR CPR only | 0.65 | 0.27 – 1.53 | 0.32 |
|  | Bystander and VCR CPR | 1.04 | 0.55 – 1.96 | 0.91 |
|  | Age | 0.98 | 0.96 – 0.99 | < 0.001 |
|  | Male gender | 0.56 | 0.35 – 0.89 | 0.015 |
|  | Home located cardiac arrest | 0.42 | 0.27 – 0.66 | < 0.001 |
|  | Time to EMS arrival | 0.94 | 0.90 – 0.98 | 0.004 |
|  | Initial rhythm shockable | 3.01 | 1.92 – 4.71 | < 0.001 |
|  | Witnessed OHCA | 2.95 | 1.85 – 4.71 | < 0.001 |

*Abbreviation*s: CPR – cardiopulmonary resuscitation; VCR – volunteer community responders; EMS – emergency medical services; OHCA – out-of-hospital cardiac arrest; OR – odds ratio; CI – confidence interval.

**Table S4**. Multivariate exact logistic regressions models for ROSC achieved (secondary outcome) for the subgroup of home-located OHCA. Model 1 is unadjusted, Model 2 is adjusted with age, gender, time to EMS arrival, initial rhythm and witness status of the OHCA.

|  | **Co-variables** | **OR** | **95% CI** | **p-value** |
| --- | --- | --- | --- | --- |
| **Model 1** | No pre-EMS CPR (reference) | 1 | - | - |
|  | Bystander CPR only | 1.38 | 0.75 – 2.54 | 0.30 |
|  | VCR CPR only | 0.61 | 0.28 – 1.32 | 0.21 |
|  | Bystander and VCR CPR | 1.07 | 0.58 – 1.97 | 0.83 |
|  |  |  |  |  |
| **Model 2** | No pre-EMS CPR (reference) | 1 | - | - |
|  | Bystander CPR only | 1.39 | 0.73 – 2.68 | 0.32 |
|  | VCR CPR only | 0.78 | 0.34 – 1.81 | 0.57 |
|  | Bystander and VCR CPR | 1.11 | 0.56 – 2.19 | 0.76 |
|  | Age | 0.99 | 0.97 – 1.00 | 0.07 |
|  | Male gender | 0.61 | 0.36 – 1.02 | 0.06 |
|  | Time to EMS arrival | 0,96 | 0.91 – 1.01 | 0.09 |
|  | Initial rhythm shockable | 2.13 | 1.26 – 3.61 | 0.005 |
|  | Witnessed OHCA | 2.96 | 1.73 – 5.07 | 0.001 |

*Abbreviation*s: CPR – cardiopulmonary resuscitation; VCR – volunteer community responders; EMS – emergency medical services; OHCA – out-of-hospital cardiac arrest; OR – odds ratio; CI – confidence interval.

**Table S5**. Multivariate binary logistic regressions model for survival to hospital admission (secondary outcome) for the subgroup of home located OHCA. Model 1 is unadjusted, Model 2 is adjusted with age, gender, home location of the OHCA, time to EMS arrival, initial rhythm and witness status of the OHCA.

|  | **Co-variables** | **OR** | **95% CI** | **p-value** |
| --- | --- | --- | --- | --- |
| **Model 1** | No pre-EMS CPR (reference) | 1 | - | - |
|  | Bystander CPR only | 1.93 | 0.96 – 3.85 | 0.06 |
|  | VCR CPR only | 0.59 | 0.23 – 1.54 | 0.28 |
|  | Bystander and VCR CPR | 1.64 | 0.82 – 3.26 | 0.16 |
|  |  |  |  |  |
| **Model 2** | No pre-EMS CPR (reference) | 1 | - | - |
|  | Bystander CPR only | 1.79 | 0.86 – 3.74 | 0.12 |
|  | VCR CPR only | 0.75 | 0.27 – 2.08 | 0.58 |
|  | Bystander and VCR CPR | 1.60 | 0.74 – 3.45 | 0.23 |
|  | Age | 0.97 | 0.96 – 0.99 | 0.0015 |
|  | Male gender | 0.82 | 0.46 – 1.46 | 0.50 |
|  | Time to EMS arrival | 0.95 | 0.90 – 1.00 | 0.07 |
|  | Initial rhythm shockable | 2.42 | 1.38 – 4.25 | 0.002 |
|  | Witnessed OHCA | 2.67 | 1.47 – 4.86 | 0.0013 |

*Abbreviation*s: CPR – cardiopulmonary resuscitation; VCR – volunteer community responders; EMS – emergency medical services; OHCA – out-of-hospital cardiac arrest; OR – odds ratio; CI – confidence interval.

**Table S6.** Comparison of the VCR network activation rate in various sub-groups according to the differences observed between the group with VCR intervention and the group without VCR intervention.

| **Subgroups** | **All cases** | **No VCR network activation** | **VCR network activation** | **p-value^*^** |
| --- | --- | --- | --- | --- |
| OHCA, n | 559 | 266 | 293 | NA |
| Home located OHCA, n (%) | 394 (70) | 153 (58) | 241 (82) | < 0.001 |
| Witnessed OHCA, n (%) | 317 (57) | 163 (61) | 154 (53) | 0.046 |
| Bystander AED usage, n (%) | 51 (9.1) | 36 (14) | 15 (5) | < 0.001 |
| Initial shockable rhythm, n (%) | 170 (28) | 65 (24) | 99 (34) | 0.02 |
| Time to EMS arrival <= 5 minutes, n (%) | 33 (6) | 22 (8) | 11 (4) | 0.04 |
| Time to EMS arrival between 5 to 10 minutes, n (%) | 176 (31) | 105 (39) | 71 (24) | < 0.001 |
| Time to EMS arrival 10-15 minutes, n (%) | 199 (36) | 80 (30) | 119 (41) | 0.01 |
| Time to EMS arrival > 15 minutes, n (%) | 151 (27) | 59 (22) | 92 (31) | 0.02 |

*Abbreviation*s: VCR – volunteer community responder; OHCA – out-of-hospital cardiac arrest; AED – automated external defibrillator; EMS – emergency medical services.

^*^The comparison between the proportion of the two groups was made using a 2-sample test for equality of proportion with continuity correction (χ^2^-test).
